# Evaluating standards for ‘serious’ disease for preimplantation genetic testing: a multi-case study on regulatory frameworks in Japan, the UK, and Western Australia

**DOI:** 10.1186/s40246-022-00390-3

**Published:** 2022-05-18

**Authors:** Kate Nakasato, Beverley Anne Yamamoto, Kazuto Kato

**Affiliations:** 1 Department of Biomedical Ethics and Public Policy, Graduate School of Medicine, Osaka University, Suita, Japan; 2 Graduate School of Human Sciences, Osaka University, Suita, Japan

**Keywords:** Preimplantation genetic testing, Disease severity, Genetic conditions, Reproductive technologies, Social implications

## Abstract

**Background:**

A number of countries are leading the way in creating regulatory frameworks for preimplantation genetic testing (PGT). Among these countries, a point of consensus is that PGT may be used to avoid the birth of a child with a serious genetic disease. However, standards for evaluating disease severity in this context are not always clear. Considering the numerous medical and social implications of defining a standard for serious disease, our study sought out to better understand how disease severity for PGT is being defined by analyzing and comparing the regulatory landscapes for PGT in various countries.

**Methods:**

We carried out a multi-case study analysis using policy documents from the UK, Western Australia, and Japan. Documentary analysis was used to analyze and compare these documents in terms of medical indications for PGT, evaluation methods of applications for PGT, and review frameworks used during the evaluation process, which includes the specific medical and social factors that are considered.

**Results:**

Within our three case studies, medical indications for PGT are based on an estimated risk of the woman giving birth to a child with a genetic abnormality with known clinical deficits. Evaluation methods for approving applications for PGT include reference to a pre-approved list of genetic conditions (the UK) and case-by-case reviews (all case studies). Review frameworks for case-by-case reviews include reference to a list of considered factors (the UK and Western Australia) and a definition statement of disease severity (Japan), which provide insight into interpretations of disease severity in each context.

**Conclusions:**

The results of this study point to the possible medical and social impacts of PGT regulatory frameworks on multiple stakeholders. Furthermore, it suggests that impacts in this case are not only caused by whether PGT is permitted or not, but also by the circumstances under which it is allowed and how decisions regarding its approval are made. Our results may serve as valuable insights for countries that already have established policy for PGT but are considering revision, countries that are without policy, and for discussions on related genetic and reproductive technologies.

## Background

### Preimplantation genetic testing

The desire to avoid suffering is a common goal across countries, cultures, and societies. It is thus no surprise that many new biomedical technologies and treatments in recent decades have focused on measures that are preventative rather than reactive, which seek to eliminate the existence of suffering before it is experienced. In terms of reproductive choices, this shift toward preventative measures has resulted in the development of reproductive and genetic technologies that can identify the possibility of a child being born with a serious genetic condition prior to their birth. The earliest developments began with prenatal diagnosis (PND) via ultrasound technology in the 1950s and later expanded to include prenatal chromosomal screening methods such as amniocentesis, chorionic villus sampling, and noninvasive tests such as serum analyte screening and cell-free DNA screening [1].

Preimplantation genetic testing (PGT) was fully developed more recently in the later twentieth century, but with initial developments beginning in as early as 1890 with embryo transfer experiments on animals. Successful attempts that resulted in human pregnancies did not occur until 1990, when PGT was used to diagnose embryos with potential genetic diseases linked to the X-chromosome [2]. PGT allows diagnostic and screening tests to be carried out on an embryo during the process of in vitro fertilization (IVF) before it is transferred to the uterus for implantation. Performing the test before implantation allows embryos with favorable traits to be selected and others to be discarded or preserved for basic research [3].

While the original purpose of PGT was to identify monogenic defects (PGT-M) that were thought to cause serious genetic diseases, applications of PGT have since expanded to include genetic screening for chromosomal abnormalities (PGT-A) and chromosomal structure arrangements (PGT-SR) as well as PGT that is used in tandem with human leukocyte antigen (HLA) typing for identifying tissue donor matches (PGT-HLA) [3–5]. The expansion of screening applications not only creates new choices for at-risk individuals to have genetically related children without passing down serious genetic conditions, but also avoids the need to terminate an affected pregnancy if it is unwanted, where this procedure is legal. This can make PGT more desirable than PND,^1^ especially in cases where terminating a pregnancy based on fetal indications is not legally permitted or goes against religious or philosophical principles [3].

Despite its potential benefits, there are also numerous ethical, legal, and social implications (ELSIs) surrounding PGT. Notable areas of concern and debate are: reproductive autonomy versus non-maleficence toward future generations [7, 8], the moral status of the embryo [9], technological risks [3, 10], increasing stigmatization and discrimination [11–13], and interfering with genetic diversity [14, 15]. Additionally, there is also the risk that PGT could be used for non-medical purposes such as elective sex-selection and preference for certain physical characteristics with known genetic links [8, 10]. In terms of regulation, the increased array of reproductive choices that PGT and other related technologies have created also bring into question who should be allowed to make these choices and the circumstances under which these choices can be made.

Further complexities arise when looking at PGT regulating policy on an international scale. While some countries, such as the UK and Australia, have created designated institutions to oversee the regulation of PGT and other reproductive technologies [9, 16], others such as Singapore delegate this responsibility to government ministries or departments of health [17]. A comparison of PGT regulatory landscapes in Nordic countries showed that despite cultural and economic similarities, there are noticeable differences in terms of which applications of PGT are allowed, if any, and whether individual cases for PGT require case-by-case evaluation by a national board of health and welfare [18]. In addition, there are countries practicing PGT such as Mexico where there are no regulations in place [19].

Previous research has also compared the basic regulatory landscapes of PGT between the USA and European countries such as the UK, France, Germany, Italy, Austria, and Switzerland [9, 16, 20]. Apart from the USA,^2^ comparisons between these countries show that a point of consensus is to limit the use of PGT-M to identify heritable genetic abnormalities in an embryo that would otherwise result in the birth of a child with a ‘serious’ genetic disease. However, the specific way of defining disease severity in this context, e.g., the explicit criteria or factors that define this standard, is not always described clearly [7, 9, 16, 23].

### Objective

To better understand how disease severity is being defined for PGT, we aimed to analyze the regulatory landscapes for PGT in Japan, the UK, and Australia using policy documents that come from the designated authoritative bodies of each country or state within a country. There were three focuses during this comparison: medical indications, evaluation methods, and review frameworks. After describing and analyzing the results, we then discuss in this paper the possible interpretations of the variations between each country and the implications of PGT on medical and social understandings of disease and genetic diversity. Finally, we conclude with a brief consideration of the significance of these results for future discussions on emerging genetic technologies within the broader context of society.

## Methods

### Inclusion criteria

This study utilized a documentary case study approach to identify the methods and frameworks that are used by designated institutions to review applications for PGT. A preliminary literature review enabled us to identify countries leading the way in regulating PGT. From these, we selected three countries that (1) have had at least 20 years of experience regulating PGT, (2) have established clearly defined institutions for carrying out regulatory efforts, and (3) have detailed policy documents that describe the circumstances under which PGT is an available option.

On top of fulfilling the aforementioned criteria, each of the three countries in this study were chosen for the following key reasons. At the time of the study, Japan’s policy on PGT was undergoing revision of its definition of a serious disease that would indicate PGT. This provided a prime opportunity to discuss policy revision within the context of PGT and the social implications of such definitions. In the UK, there is a list of pre-approved conditions for PGT that is referenced during decision-making processes. Currently, there are over 600 conditions on this list.

The distribution of regulatory power was another factor in the data selection process. Countries such as the USA and Canada could not be included due to the complexity of their regulatory landscapes that is caused by decentralization in the distribution of power over PGT; however, Australia was the exception. The distribution of power over PGT is clearly defined, and unlike the USA and Canada, detailed policy is available at both the state and federal level. Including Australia in the study thus allowed us to consider how a country with a decentralized distribution of power over new reproductive technologies may regulate PGT. Due to the autonomy of each state to define its own policy in reference to federal guidelines, we chose to investigate Western Australia based on the level of detail of its policy, compared to the other states.

Within the selected countries, we identified the institutions with jurisdiction over PGT based on their ability to create policy as well as monitor and license reproductive clinics that carry out PGT. Documents that were gathered from these institutions included acts/laws, regulations, guidelines, reports, opinion statements, and documents meant to inform the public. The timeframe for documentary inclusion was from 1990 to October 2021.

### Comparisons

We focused on three areas for comparison: (1) medical indications, (2) evaluation methods, and (3) review frameworks. Medical indications refer to the specific circumstances under which PGT is allowed. Evaluation methods are the processes through which the designated institution decides whether the individual circumstances of those seeking PGT meet their standard for disease severity. Review frameworks refer to the specific decision-making tools that are used during case-by-case review. They also include the specific medical or social factors that are considered during the review, which are thought to constitute the standard for disease severity.

This study also focused specifically on recent policy revisions that have taken place in Japan over the past 2 years regarding the definition of a serious disease in terms of PGT. Using records from public forums that documented the revision process, we investigated the details regarding background motivations, new standards that this change sets forth, and how the resulting regulatory landscape compares against the other two countries in this study. Analyzing Japan’s revision process not only highlights the continuously evolving nature of the regulatory landscape of PGT, but also suggests a possible direction in which future understandings of disease severity may be moving.

## Results

The following subsections describe the regulatory landscape of PGT in the UK, Western Australia, and Japan. The first three subsections summarize the distribution of power over PGT in each country, respectively, in terms of the institutions and policy documents that can influence who may utilize PGT and under which circumstances. They also briefly summarize the medical indications for PGT, evaluation methods of PGT applications, and the review frameworks. The latter three subsections look at comparisons within each of these areas in greater detail.

### UK

PGT in the UK is regulated by the Human Fertilisation and Embryology Authority (HFEA). It was established by the Human Fertilisation and Embryology Act in 1990 [24] and oversees licensing and monitoring of clinics. All clinics and research centers dealing with human embryos need to be licensed under the HFEA and must comply with its rules and regulations [25]. Foundational laws regarding clinical application of PGT are laid out in the HFE Act. It states that PGT may be used when there is a risk that the child will be born with a serious disability, illness, or other hereditary medical condition [24]. More specific details regarding the application process can be found in the HFEA Code of Practice [26] and on online web pages oriented toward informing the public about treatment options [27]. In these documents, the HFEA establishes clear guidelines for both the designated testing centers and the public. However, the HFEA does not play a direct role in the decision-making process of each individual case. Instead, the HFEA maintains a list of over 600 conditions that have been pre-approved for PGT in general. This list is easily accessed from the HFEA’s online webpage [27] and serves to inform both the testing centers that are licensed to carry out PGT as well as members of the public who may be considering PGT as an option. If the condition for which PGT is to be used is on the list, those seeking PGT can be referred to a regional clinical genetics service by their general practitioner. The final decision on the appropriateness of the procedure is made through discussion between designated physicians at the testing center and those seeking treatment. During this decision, the testing center must consider a set of medical and social factors as designated by the HFEA Code of Practice [26]. The HFEA itself is not directly involved in each individual application for PGT unless it is regarding a condition that has not yet been added to their pre-approved list. In this case, the designated testing center makes an application to the HFEA on behalf of those seeking treatment. The HFEA then refers to the aforementioned set of medical and social factors to decide whether the condition should be added [26]. In other words, the HFEA’s pre-approved list serves as the primary foundation on which decisions are made.

### Western Australia

PGT in Western Australia is regulated broadly at the national level by guidelines from the National Health and Medical Research Council (NHMRC) [28]. At the state level, PGT falls within the jurisdiction of the Reproductive Technology Council (RTC). The RTC has the power to establish rules of practice and provide recommendations to the Western Australia Department of Health [29]. Like the UK case, foundational laws regarding PGT in Western Australia were laid out by the Human Reproductive Technology Act in 1991. In regard to PGT, section 14(2b) states that ‘the [RTC] may not grant approval to any diagnostic procedure to be carried out upon or with a human embryo unless… where the diagnostic procedure is for the genetic testing of the embryo, there is a significant risk of a serious genetic abnormality or disease being present in the embryo’ [30]. Further details are specified by the RTC’s guiding policy document on embryo-related procedures, the Policy on Approval of Diagnostic Procedures Involving Embryos [31]. An online informational pamphlet aimed at informing the public also exists to provide additional information [32].

Each individual PGT application must be approved by the RTC on a case-by-case basis. Decisions are made based on a set of medical and social factors relating to the individual circumstances of those seeking treatment. The factors that must be considered during these decisions are designated by the RTC under the guidance of the NHMRC’s suggestions [31]. Maintaining a pre-approved list of possible genetic conditions for which PGT may be utilized does not exist, as it has been pointed out that ‘it is not possible to list the genetic conditions, diseases or abnormalities for which the use of PGT is ethically acceptable, as context is important and the assessment may change over time’ [33].

### Japan

PGT in Japan is overseen by the Japanese Society of Obstetrics and Gynecology (JSOG). While the JSOG does not have explicit legal power, clinics that use reproductive technologies are obliged to follow the JSOG’s guidelines. Failure to do so may result in a loss of membership and therefore the ability to practice reproductive medicine [34, 35]. The JSOG made its first statement on PGT in October 1998, by stating that PGT was permitted but limited to testing for ‘serious’ disease only. An ethics subcommittee was created to evaluate the circumstances of those seeking treatment on an individual case-by-case basis [36]. Until recently, guidelines for PGT stated that a serious disease was one that significantly impairs the daily life or threatens the survival of the child before reaching adulthood. Significant impairment to the daily life of the child was understood as a severity level where the said child was unable to sustain life without use of a ventilator. Based on this definition, additional factors that were taken into consideration include the penetrance rate (likelihood that the genetic abnormality will manifest as clinical symptoms), the expected age of onset, and the severity of symptoms, the number of family members that have the condition and the level of severity of their symptoms, and the possibility of treatment [36, 37].

Policy revisions for the standard for disease severity in Japan began after the JSOG received an application in 2019 from a patient with retinoblastoma seeking PGT, whose condition did not meet the interpretation of a serious disease at the time, but from other perspectives could be thought to significantly impair the daily life of the child [36, 38]. The inherited form of retinoblastoma follows an autosomal-dominant inheritance pattern, meaning only one parent needs to be a carrier for there to be a 50% chance of passing on the defective gene to their offspring. Early detection is crucial for effective treatment, and as such genetic testing may be recommended to determine the child’s risk of developing this condition [39]. Various concerns were voiced during the review process of the patient’s application, the two strongest ones being (1) that part of the standard for disease severity was based on whether age of onset was before adulthood and (2) whether it was sufficient enough for the debate on the seriousness of the retinoblastoma case to be carried out by a committee that was composed of only medical doctors, rather than one that included experts from diverse academic fields to provide broader perspectives from ethical and social viewpoints [36]. Although some argued that retinoblastoma was serious enough due to its impact on the daily life of the future child, opposing concerns were raised that if PGT were to be approved for this case, the use of PGT for non-life-threatening conditions would increase [36, 38].

The dilemma of the retinoblastoma case prompted the JSOG to reflect on the fact that the current standard for disease severity for PGT was based only on medical criteria. As a result, it was seen as necessary to revise the standard while considering a broader range of factors that contribute to the lived realities of genetic diseases and conditions. Included in discussions during the revision process were a diverse range of participants that included medical, humanities, and social science professionals as well as patient groups^3^ and members of the public. The meetings were also open to online viewing and to commentary from the public [36].

Underlying the discussions was the awareness of the potential negative social impact that PGT can have on the lives of people currently living with genetic conditions. Stakeholders that were against expanding the applications of PGT by removing the ‘before adulthood’ indication voiced that such a revision, e.g., opening the possibility for including adult-onset diseases, would make it more difficult for people currently living with genetic conditions to have fulfilled lives [40]. It was stated that the goal moving forward should be to strive toward a society where all people, whether they have a disability or not, can live healthy lives (*shougai ga aru kata mo nai kata mo, dare mo ga kenkou ni ikirareru yo no naka;* 障碍がある方もない方も、だれもが健康に生きられる世の中) and that moving forward, there is a need to sustain multidisciplinary discourse through the collaboration of experts of the medical field, ELSI disciplines, individuals and families who have been affected by the genetic condition in question, and patient groups [37]. The final report on this matter sets the standard for disease severity as the following:

[A serious disease in this context is,] as a general principle, a condition that causes symptoms that strongly impair daily life or threaten the survival before reaching adulthood, and for which there is no effective treatment to avoid such symptoms, or for which highly advanced and invasive treatment is necessary [36].

Although discussions had been moving toward a consensus about removing the indication of age of onset of ‘before reaching adulthood,’ ultimately the committee decided to leave it in based on the thought that the JSOG should not promote PGT, but rather act based on the individual circumstances of each case [36]*.* At the same time, considerations for conditions that manifest after adulthood resulted in the following addition to the statement:

When making a judgement on a case for which there has been no review experience, it is necessary to request the opinion of an expert group (clinical or genetic) … where the opinion is based on a medical perspective… but also takes into consideration the lifestyle background and thoughts of the couple seeking treatment [36].

In other words, the discussions resulted in a form of compromise. The new definition does not completely open up PGT for all conditions regardless of age of onset; however, the addition of ‘as a general principle’ (*gensoku to shite;* 原則として) provides the flexibility to make exceptions under certain circumstances. Furthermore, in the case that an application is declined based on the opinion forms submitted by the expert groups indicated above, it is possible for those seeking treatment to re-apply and be evaluated by a clinical ethics review committee, initiated by the JSOG, that is comprised of diverse stakeholders such as members of patient groups and non-experts from the public.^4^ The revision thus suggests a shift toward more inclusive policy-making and greater awareness of the diverse circumstances related to living with a genetic condition.

### Medical indications

For all cases, the term *serious* (*juutoku;* 重篤 in Japanese JSOG policy) is used to describe the circumstances under which PGT may be applicable (Table 1). This is consistent with previous literature that has described the status of PGT in various countries [7, 9, 16, 23, 35]. An additional point of similarity is the specification that the targeted genetic or abnormality should be one that manifests as clinical symptoms in the child. This specification is important because it indicates that the target that PGT is meant to avoid is not the genetic abnormality itself, but rather the symptoms, impairment, or suffering with which the genetic abnormality is associated in children. At the same time, each case shows subtle differences. Western Australia explicitly states that a ‘genetic abnormality or disease in the embryo is not simply a defect in the genetic material, but is one associated with a known clinical deficit’ [31]. While this is implied in the indications of the UK and Japan, Western Australia shows explicit clarification. Additionally, the UK does not use the term *disease.* Instead, it is substituted for *disability, illness,* or *medical condition.* This wording is in-line with the preferred language of disability groups [41]*.* The UK is also the only jurisdiction in this study to include mitochondrial abnormalities.

**Table 1:** Medical indications

UK	Western Australia	Japan
1. Genetic abnormality 2. Chromosomal abnormality 3. Mitochondrial abnormality …that will manifest as a… 1. Serious physical or mental disability 2. Serious illness 3. Serious medical condition	1. Serious genetic abnormality 2. Serious genetic disease 3. Serious genetic condition …that is associated with a known clinical deficit	1. Genetic mutation 2. Chromosomal abnormality …that may result in the birth of a child with a serious genetic disease

### Evaluation methods

Each jurisdiction in this study employs a different evaluation method when handling applications for PGT. Evaluation methods refer to the combination of decision-making frameworks that are utilized by the designated institution to approve or reject PGT applications. These may include a pre-approved list of conditions, case-by-case review, and the more specific review frameworks that are employed during any case-by-case review (Table 2).

**Table 2:** Evaluation methods

	UK	Western Australia	Japan
Pre-approved list of conditions	Yes	No	No
Case-by-case review by designated institution	Yes** Only to approve conditions not already on list Otherwise carried out by designated testing center	Yes	Yes
Review framework	List of factors	List of factors	Definition statement on disease severity

The evaluation methods in Japan and Western Australia are similar in the sense that they both rely mainly on case-by-case evaluation by the designated institution. In Japan, the JSOG uses an overarching definition statement to set the standard for a serious disease, the risk of which would indicate PGT. The statement itself emphasizes the main factors that should be considered, while a short list of additional factors is noted separately. Based on these factors, the JSOG approves applications from those seeking treatment on a case-by-case basis.

In Western Australia, the RTC utilizes a list that includes both medical factors, which are based on the profile of the genetic condition to be tested for, and social factors, which are based on the individual circumstances of those seeking treatment. These factors are divided into hierarchical distinctions, where some are labeled as ‘essential’ to the evaluation process, while others are labeled as ‘desirable.’

Unlike the previous jurisdictions, the HFEA in the UK maintains a list of over 600 pre-approved conditions that are thought to be serious enough for PGT. Under the circumstances that the HFEA must review an application for a genetic condition that has not yet been added to the list, their review framework is based on a list of medical and social factors, like the RTC in Western Australia. However, there is no distinction between factors that are essential versus those that are desirable. Furthermore, due to the significant volume of conditions that are already on the pre-approved list, it is more likely that the final decision will be left to the physician at the designated testing center of those seeking treatment. To the best of our knowledge, the UK is the only country to utilize a pre-approved list of genetic conditions for PGT.

### Review frameworks

Further differences were identified within the specific medical and social factors that are indicated by the review frameworks of each evaluation method (Tables 3, 4). Common factors across all three jurisdictions in this study are the medical factors which are related to the potential impact on the daily life of the child and the availability of treatment for the condition. Review frameworks by the HFEA and RTC include both medical and social factors (Table 3). In these cases, it is common to refer to the support that would be available for the future child and the views of the family. Factors that are included in the review framework of the JSOG are noticeably fewer, yet broader than those of the HFEA or RTC. This is likely due to the fact the factors are embedded within an overarching definition statement on disease severity rather than being presented in a list format. Compared to the other two institutions, the JSOG appears to put a particular emphasis on the impact a condition will have on the everyday life of the child, i.e., the quality of life (QOL). The definition statement itself refers only to medical factors. Social factors are indeed considered as well; however, they are indicated as supplemental factors or factors to be considered in exceptional cases (Table 4).

**Table 3:** Review frameworks in the UK and Western Australia

List of factors	
UK	Western Australia
1. The views of the people seeking treatment in relation to the condition to be avoided, including their previous reproductive experience 2. The likely degree of suffering associated with the condition 3. The availability of effective therapy, now and in the future 4. The speed of degeneration in progressive disorders 5. The extent of any intellectual impairment 6. The social support available 7. The family circumstances of the people seeking treatment [26]	*Essential* 1. Is there a significant risk of a serious genetic abnormality or disease in the context of the family that is requesting the testing? 2. What is the genetic abnormality or disease that is to be tested for? 3. What experience with, and attitude to, the abnormality or disease does the family requesting the testing have? 4. What factors indicate that there is a risk that the embryo will be affected by the genetic abnormality or disease? 5. What is the level of impairment to body functions and structures that is usually associated with the abnormality or disease? 6. What difficulties would a person with the abnormality or disease be expected to have in participating in activities such as learning and applying knowledge, communication, mobility, self-care, employment and community, social and civic life? *Desirable* 1. What is the level of support that would be required by a person who has the abnormality or disease? 2. What are the prospects for new and longer-term treatments and interventions for the condition? 3. What is the capacity of the family who are requesting the testing to provide the level of support required by a child with the abnormality or disease? 4. What clinical genetic and diagnostic data are to be used in the testing procedure? 5. What other testing options are available? 6. What level of information will be possible from the test, in terms of interpretation, sensitivity and specificity (includes error)? 7. Has the person requesting the testing been provided with counselling about the potential impact of testing and contact information for other persons or organizations that have experience with the condition? [31]

**Table 4:** Review framework in Japan

Definition statement	
Previous (2016)	A serious disease is characterized by… 1. Symptoms that *strongly impact daily life* before adulthood 2. Symptoms that *threaten survival* before adulthood
Revised (2021)	A serious disease is characterized by symptoms that, in principle… 1. *Strongly impact daily life* before adulthood; 2. *Threaten survival* before adulthood; And in the case where treatment for the condition is… 1. Not available; 2. Available but highly advanced and invasive
Supplemental factors	1. Penetrance of symptoms 2. Predicted age of onset of symptoms in the child 3. Predicted degree of severity of symptoms in the child 4. Number of family members that have the condition 5. Degree of severity of symptoms of family members that have the condition 6. Possibility of treatment
Exceptional cases	Regarding cases with which the JSOG has had no previous review experience, an opinion form from an expert organization (clinical or genetic) must be submitted, which includes… 1. Evaluation from a medical perspective (accuracy of the test, standard of disease severity) 2. Consideration of the circumstances and opinions of those seeking treatment

## Discussion

In this section, we discuss the societal impacts of the regulatory frameworks that have been described in this study. It is divided into three parts. First, we focus mainly on the labeling of genetic conditions that can occur as a result of having a pre-approved list of conditions for PGT. Second, we discuss case-by-case review and the importance of including non-medical factors in the decision-making process. Finally, we briefly discuss how insight from patients living with genetic conditions can be incorporated into the decision-making process. The purpose of these discussions is not to promote one country’s approach over another, but rather to compare their contextual advantages and potential impacts on society.

### Pre-approved lists and labeling

Our results show that, according to the regulation for PGT in the three countries of this study, PGT may be used to test for a genetic abnormality that will likely result in the birth of a child with a serious genetic condition. When decisions are made based on the estimated level of severity, a label of seriousness is created. Where this label is placed and how strongly it affects those who are receiving testing, however, depends on aspects of the regulatory framework.

Referencing a pre-approved list requires explicitly naming certain conditions for which PGT may be used. This places the initial judgment, and thus the label of seriousness, on the genetic condition itself. As a result, when decisions whether to carry out PGT are made between the designated physician and those seeking treatment, the label of seriousness has already been pre-assigned to the condition in an explicit manner. The conditions that are considered serious enough for PGT are made clear from the beginning, based on medical indications. The consideration of non-medical factors based on individual circumstances is made subsequently. On the one hand, this can make the decision-making process clearer and more straightforward. Confusion surrounding the standard for disease severity may be avoided, which is especially important in situations where abortions based on fetal indications are not allowed. PGT becomes a crucial aspect of reproductive autonomy, and decisions on applications for PGT that are appropriate and timely are extremely important for those with serious genetic conditions who may have the chance to access this technology. The debate in Japan surrounding the patient with retinoblastoma highlights this fact, as abortions based on fetal indications are not allowed in Japan.

However, there are several negative impacts that can occur by placing the label of seriousness on the condition itself. By doing this, a medical authority is placed on the choice to utilize PGT to avoid the birth of a child with a serious genetic condition, and as a result an accountability for being born emerges [12, 42]. Parents of children who are already living with a condition that has been labeled as serious, as well as the children themselves, may feel the need to justify having been born despite the availability of PGT or other related tests [42]. Labeling a condition as serious may also worsen the social stigma surrounding it, which in turn may influence the perceived impact of the condition on the QOL of the child who would be born [33]. Amendments in the form of removing a condition from the pre-approved list may cause confusion among the public and distress for those who would lose their ability to access PGT based on these changes. Finally, pre-assigning the label of seriousness on the genetic condition itself may suggest that all cases of the same condition are experienced in the same way, which does not account for the diverse lived realities of genetic conditions.

Similar challenges can be seen by another example of making medical decisions based on labeling and explicitly assigning medical conditions into certain categories. In 1985, guidelines published by a Japanese physician described a classification system for deciding whether to provide intensive care to newborn infants with disabilities. These guidelines are popularly known as ‘Nishida’s guidelines’ and were first published in 1985, with periodic revisions based on ethical and social considerations. They designated four different classes of conditions and the appropriate intensive care measures for newborn infants born with these conditions.^5^ In an attempt to clarify the class under which an infant should be treated, these guidelines initially cited the names of specific conditions for some of the categories. This, however, received notable criticism and debate. There were difficulties regarding the change of certain conditions from one class to another, and it was pointed out that explicitly naming certain conditions did not fully consider the diverse lives that children born with the same condition may have [43]. Health care centers in Japan have since made their own guidelines. However, Nishida’s guidelines were the first of their kind in Japan and have had significant influence on not only the guidelines that followed it, but also the families and children whose care fell under its guidance [43]. This example not only emphasizes the challenges of having a pre-approved list for PGT, but also highlights the direct societal impacts of placing labels on medical conditions.

### Case-by-case evaluation and non-medical factors

We now return to the subject of PGT and move on to discuss case-by-case review and the importance of including non-medical factors in the decision-making process. Case-by-case review without referencing a pre-approved list avoids directly labeling the condition itself and takes a more holistic perspective by simultaneously considering medical indications as well as non-medical factors based on the individual circumstances that are unique to each case. The label of seriousness is placed on the case as a whole rather than pre-assigning it to the condition beforehand. The condition itself may be indirectly labeled as serious based on the ultimate decision to approve or reject its case for PGT; however, this may not have the same social impact as using a pre-approved list. Case-by-case review also allows for flexibility to consider diverse lived experiences of genetic conditions. This is important because even when standards of disease severity for PGT are rooted in objective medical indications, the lived realities of a genetic condition can vary significantly, even among individuals with the same condition. These variations may occur based on how the symptoms of a condition manifest,^6^ how the individual relates to their condition,^7^ and how they are treated in society^8^ [42]. It is the combination of these dimensions that contribute to each individual’s experience living with their genetic condition, which cannot be adequately predicted based solely on medical factors.

### Patient insight

A crucial aspect of understanding the lived experiences of genetic conditions is the direct insight from patients and their families, which we discuss in the following two aspects. First, we recognize the benefits of direct contact between those seeking PGT and patients living with genetic conditions. Second, we consider the implications of the involvement of patient stakeholders in the policy-making process for PGT.

During case-by-case evaluation, the RTC considers whether those seeking PGT have been provided with the contact information of other patients or organizations that have experience with the condition. This practice of directly connecting those seeking PGT and patients with genetic conditions may remove the filter that would be created when the insight from these patient stakeholders is conveyed through the perspective of a medical professional. It creates a space for direct and personal dialogue between those seeking treatment and patient stakeholders. This step in the application process is not indicated by the HFEA or the JSOG.

A notable aspect of the situation in Japan, however, is the JSOG’s inclusion of patient groups during the meetings for revising the disease severity definition. This embeds patient insights into the policy-making process. Placing the role of patient insight at an earlier stage during the policy-making process itself provides an opportunity for the diversity of the lived experiences of genetic conditions to be addressed at a systematic level. It also strengthens patient involvement, which can contribute not only toward more appropriate policy, but also patient empowerment.

## Conclusions

The results of this study point to the possible medical and social impacts of PGT regulatory frameworks on multiple stakeholders. Furthermore, it suggests that impacts in this case are not only caused by whether PGT is permitted or not, but also by the circumstances under which it is allowed and how decisions regarding its approval are made. Our results may serve as valuable insights for countries that already have established policy for PGT but are considering revision, countries that are without policy or are currently in the process of policy-making, and for discussions on related genetic and reproductive technologies. The importance of considering the direct impacts of PGT policy is perhaps even greater in countries with significant societal or health inequalities, as they may be further exacerbated if prudent oversight of these technologies is not in place. Although the scope of this study is limited to only three countries, we believe that it adds an additional layer to the discourse regarding interactions between emerging genetic technologies and society.

## Data Availability

The data that support the findings of this study were derived from the following resources available in the public domain: reference numbers 22, 26, and 27 (the UK), 30, 31, 32, and 33 (Western Australia), 35, 36, 37, 38, and 40 (Japan).

## Abbreviations

PGT: Preimplantation genetic testing
PND: Prenatal diagnosis
IVF: In vitro fertilization
PGT-M: Preimplantation genetic testing for monogenic/single defects
PGT-A: Preimplantation genetic testing for aneuploidy
PGT-SR: Preimplantation genetic testing for structural rearrangements
HLA Typing: Human Leukocyte Antigen Typing
HFEA: Human Fertilisation and Embryology Authority
HFE Act: Human Fertilisation and Embryology Act
NHMRC: National Health and Medical Research Council
RTC: Reproductive Technology Council
HRT Act: Human Reproductive Technology Act
JSOG: Japan Society of Obstetrics and Gynecology
QOL: Quality of life

## Footnotes

1 Similarly, PGT is sometimes included in discussions related to the utilization of gene therapy. Currently, the safety and efficacy of PGT is more established than that of gene therapy. It may be possible that advances in gene therapy will make it a feasible or even desirable alternative in the future. However, whether it would be a superior alternative to PGT would still depend on individual circumstances. Gene therapy may be more desirable in cases where the manipulation of a human embryo is not legally or morally permitted. Moreover, PGT is not possible in cases where an at-risk individual seeking treatment is homozygous for an autosomal-dominant condition (e.g., Marfan syndrome). On the other hand, PGT may be more desirable when a genetic condition does not allow a newborn to survive past infancy or affects tissue in the body that gene therapy cannot effectively reach (e.g., Huntington’s disease) [6]. These diverse circumstances alert us to the importance of decision-making on a case-by-case basis as genetic and reproductive technologies continue to advance, and more options become available.

2 There are no explicit state or federal regulations for PGT in the USA. The safety and efficacy of the technology itself is regulated by the Food and Drug Administration (FDA), but *when and how* the technology is used is subject to the discretion of the designated physician. Academic organizations such as the American Society for Reproductive Medicine (ASRM) have published basic information on the possible applications of PGT, but they are significantly less detailed than the national guidelines from other countries such as the UK and Australia [21]. A 2018 study showed that 93.6% of artificial reproductive technology (ART) clinics in the USA offer PGT for non-medical sex-selection [22].

3 Some of the participating patient groups included the Myotonic Dystrophy Patients’ Group of Japan, the Peer Support Group of Retinoblastoma (RB Peer Support Group), the Association for the Future of ALD (adrenoleukodystrophy), Japan Huntington’s Disease Network, and so on [36].

4 The composition of the committee must meet the following requirements: (1) only two members belong to the JSOG’s PGT subcommittee (the chairperson and one other committee member), (2) members include experts in medicine, natural science, ethics, law, humanities, and social science, someone who can provide an opinion from the perspective of the research participant from a general standpoint, both members and non-members of the JSOG ethics review committee, both men and women, (3) the committee is made up of at least five members [36].

5 Class A: maximum therapeutic effort; applies to most infant patients. Class B: selective therapeutic procedures; applies to infant patients whose life prognoses are known to be short such as epidermolysis bullosa and congenital myopathy. Class C: general nursing care (protection, nutrition, wiping, affection) that does not go beyond current treatments; applies to trisomy 13, trisomy 18, infants born less than 500 g, and infants with severe paralysis, intracranial hemorrhage, on artificial ventilation, etc. Class D: ceasing of all medical treatment. The relevance of Class D varies based on the individual hospital in Japan. Nishida’s guidelines were originally based on Duff and Campbell’s 1973 class designations of a similar nature [43].

6 Variations may occur in terms of the likelihood of whether the genetic abnormality manifests as clinical symptoms, the age of onset, progression, stability, and suffering experienced by these symptoms, and the individual’s response to treatment [44].

7 Based on the way a condition manifests, a condition may be seen as either a disruption to one’s life or as a part of their identity [42, 45]. It is likely that an onset of symptoms that occurs comparatively later in life, a progressive deterioration of the condition, and symptoms that cause significant suffering for the individual are more strongly associated with the externalization, rather than identification with one’s self, of the condition [42].

8 According to the social model of disability, a distinction can be made between the suffering that comes from the physical or mental impairment that is directly caused by condition versus the suffering that comes from disadvantages, discrimination, and stigmatization that is caused by certain institution in society [11, 13, 42].
